# Novice Doctors in the Emergency Department: A Scoping Review

**DOI:** 10.7759/cureus.26245

**Published:** 2022-06-23

**Authors:** Patricia Stassen, Dewa Westerman

**Affiliations:** 1 Internal Medicine, Department of General Medicine, Section of Acute Medicine, Maastricht University Medical Centre, Maastricht, NLD; 2 General Practice, Maastricht University, Maastricht, NLD; 3 Optimising Patient Care, Care and Public Health Research Institute, Maastricht, NLD; 4 Health Professions Education, Maastricht School of Health Professions Education, Maastricht University, Maastricht, NLD

**Keywords:** organization, preparation for future learning, preparedness training, emergency department, novice doctors

## Abstract

In many emergency departments (EDs), young, inexperienced doctors treat patients who are critically ill. At the start of their career, these novice doctors are not sufficiently qualified to take care of these potentially critically ill patients in the highly demanding environment of an ED. This not only poses a threat to the well-being of the doctor, who feels inadequately prepared and experiences a lot of stress, but also to that of the patients, who may not receive optimal care. Lastly, young doctors may influence the efficiency of the organization, with longer throughput times, more orders of ancillary investigations, and more admissions.

Training novice doctors with regard to simple or complex skills using simulation techniques is part of the solution. However, the transfer of newly learned skills to clinical practice remains unexplored, and not everything can be trained before the actual skill is required. Therefore, it is important to train young doctors in their learning abilities, for instance, teach them how to be adaptive and how to use their skills in new situations. Lastly, the way care is organized is essential. Good supervision, leaving room for the learning processes of young doctors, developing a team with more experienced professionals (paramedics, nurses, and doctors), and well-organized processes, aiming to reduce the complexity of the work, are ways to improve the quality of care, independent of the experience level of the novice doctor.

## Introduction and background

The pressure on emergency departments (EDs) is generally found to increase over the years [1], and so are the demands on the doctors who work in these departments [2]. During their work in assessing and treating potentially critically ill patients with increasingly complex problems, doctors are frequently interrupted [3,4] by colleagues and other personnel or telephone calls [5]. Besides, this multi-tasking occurs in a noisy environment [6]. Often, in addition to taking care of patients, busy doctors have to teach junior colleagues and interns.

Doctors who work in this highly demanding environment require a substantial number of competencies, which can be categorized into technical and non-technical skills. Technical skills include resuscitation techniques, performing physical examinations, and interpreting X-rays. Non-technical skills include dealing with diagnostic uncertainty, making decisions based on incomplete information, working in teams, and developing communication skills [7-9].

The number of required competencies is high, and these take considerable time to acquire. The question, therefore, arises whether young, inexperienced doctors have sufficient tools to handle the high load of potentially critically ill ED patients.

In this scoping review, we focus on the problems that novice doctors in the ED have to face as well as their consequences. Furthermore, we will elucidate the possible ways to overcome these problems.

## Review

Novice doctor perspective

The feeling of being in control, confident, and prepared is necessary to perform well, be adaptive, and learn from new situations. However, novice doctors often feel inadequately prepared when they start working in the ED [10-15]. In addition, novice doctors stationed in the ED experience more anxiety and less job satisfaction than those stationed elsewhere in the hospital [16]. This feeling of inadequate preparation and the resulting anxiety and dissatisfaction may increase as, over the years, the competency in acute care of novice doctors is declining [14].

Young doctors need to learn their skills in a short frame of time. These include transferring their knowledge into practice, making decisions while not being sure about the situation and under time pressure, adapting to a new stressful culture with its specific hierarchy, and bearing a new responsibility [9].

When novice doctors start in their new roles, we should be aware that their thinking process differs from that of experienced doctors. Differences in cognitive handling between inexperienced and experienced doctors have indeed been identified [9]. Novice doctors were found to overly rely on objective data and to make a diagnosis quickly but were not able to take data that did not fit within their conclusion into account [17]. In contrast, experienced doctors were able to adapt their conclusions to new or aberrant information and were able to overview the big picture.

Novices use their working memory (i.e., process information) differently than experts. With increased expertise, doctors develop more complex and inclusive decision-making strategies [17] and are able to extend their long-term working memory. This difference means that for novice doctors, it takes more mental effort to perform a certain task than for experienced doctors [18].

Experienced doctors are able to develop the ability to intuitively know what to do and to quickly recognize critical aspects of a situation [19]. This intuition is generally developed through experience. More experienced physicians need fewer contextual cues and ancillary investigations to verify, and be certain of, the diagnosis. Indeed, fewer risk aversion strategies (e.g., ordering ancillary tests) were noted among experienced doctors [20]. These findings fit in the naturalistic decision-making theory stating that experienced doctors mostly use past experiences and pattern matching to make decisions [21].

Recognizing patterns allows experienced doctors to reach a provisional diagnosis more quickly and enables them to deal with their lack of certainty. One of the consequences of the lack of experience is that it takes more time to assess and treat acute patients [20].

One important factor that impacts cognitive performance and the ability to multitask is the stress level. This multi-tasking, frequently required in the ED, adds to the stress novice doctors experience [22].

Patient perspective

Under-preparedness is not only stressful for young doctors [10] but also impacts the quality of care and could be a patient safety issue [23,24]. For specific skills, such as the interpretation of chest X-rays [25] and electrocardiograms [26], suboptimal accuracy has been found in novice doctors. In addition, procedural skills were found to be not sufficient [27].

The gaps in experience, knowledge, and skills influence the capacity of the novice doctor to manage deteriorating patients [12,23] and lead to poorer outcomes for ED patients. Patient outcomes in the period after the changeover of novice doctors were found to be worse with an increase in patient mortality of 5% [24]. Likewise, in another study, higher mortality was found during weekends, which was linked to the employment of inexperienced doctors [28].

Organizational perspective

The employment of novice doctors has an impact on the efficiency of the ED. Following the yearly rotational shifts, during which doctors are replaced and teams are changed, decreased efficiency of care was found [24]. Examples of inefficiency are a higher number of admissions to the hospital and ordered advanced imaging and longer ED stays when novice doctors work in the ED [29-31]. The associated costs may be charged to the patients depending on the way healthcare is financed.

The ED can be considered a high-risk place in a medico-legal way as well. The number of, very expensive, claims is increasing [32], and these claims often result from the way residents are employed and supervised [33].

Possible solutions

Looking at all the evidence showing that novice doctors are underprepared, not happy, and performing suboptimally, one could mistakingly conclude that these young doctors should not be employed in the ED. On the contrary, the conclusion should be that the way we employ these novice doctors in the ED is suboptimal. Interestingly, the best way to do this has not been (extensively) investigated [11]. The solutions we found feasible can be divided into two categories: better preparation and training, and a better organization of the ED.

Preparation and training

As a first step, it should be acknowledged that working in the ED is highly demanding and requires a considerable number of skills. This means that proper preparation is crucial. The second step is to identify the competencies (i.e., knowledge, technical, and non-technical skills) that are required. For residents working in the field of surgery, these requirements will differ from those working in the field of internal medicine. Involving ED nurses in defining the competencies needed in the ED provides useful insights [34].

The requirements regarding knowledge should focus on the problems that are frequently encountered in the ED, for instance, protocols on sepsis or pain management. A list of topics can be composed for each specific domain. Familiarization with these topics and particular protocols may relieve the cognitive load of the novices and increase their self-confidence [35].

The requirements regarding skills include domain-specific physical examination skills and procedures. These can be taught as a single task, such as ascites drainage, but also in the context of other tasks. The ABCDE methodology and resuscitation skills are examples of complex skill tasks.

Teaching novice doctors in a simulated setting is highly popular. It is indeed proven that these simulation courses improve the confidence and performance of doctors [23,36]. However, there seems to be a gap in the transfer of the skills learned in a simulation lab to the real practice of a busy ED. In one study, only in a minority of potentially unstable ED patients, the ABCDE methodology was applied [37], and in another study, many residents felt inadequately prepared despite having received a targeted training before their first shift [15]. This means that providing courses in a simulation lab is not enough. It seems necessary that novice doctors are observed and provided feedback in real practice regarding the way they use and perform the ABCDE methodology (and other skills). The efficacy of simulation may further be improved by creating the most realistic working environment possible [14]. Courses on teamwork and crisis resource management [38] are effective in improving team performance [7,17,39]. In addition, they reduce the stress levels of novice doctors [40].

The list of competencies that need to be acquired is, however, becoming longer and longer. Newly identified competencies such as situational awareness [41] and multi-tasking [4] are examples. Instead of making the list longer and adjusting curricula, maybe we should focus on preparing our novice doctors for future learning [42-44]. Being prepared for future learning means that novice doctors have acquired the ability to constantly learn from new situations. Following this concept, instructors use a different approach, “discover-then-tell” instead of “tell-first,” aiming to activate knowledge that is already present and promoting the doctors to discover ways of comprehending and solving a problem. Currently, the first studies investigating the effect of applying the principles of preparation for future learning have been published [43,45].

Organization

Another important step is the optimization of the staffing of the ED. First, staffing the ED with senior physicians who can provide adequate supervision of novice doctors is necessary. This staffing with experienced doctors improves both the outcome for the patients and the efficiency of care [46]. A reduction of admissions of 11% was seen in one study when senior doctors supervised novice doctors [47]. Adequate supervision should at the same time serve the novices, guiding them during their first steps in emergency medicine, leaving room for individualization, autonomy, and entrustment [11,46,48]. Adequate supervision includes direct observation of residents, which in practice is unfortunately not often done [49].

Second, keeping in mind that novice doctors who are placed in the ED experience more stress than those placed in other wards, it seems sound to only place doctors with some experience in the ED. By doing this, the novice doctors will have made progress in their experience and their technical and non-technical competencies. Then, employment in the ED will be less stressful and there will be more room for being adaptive and learning in the ED. Since 2022, in the Netherlands, at least one year of relevant experience is required for doctors working in the ED [50]. As an alternative or additional solution, novices shadow more experienced doctors during the first weeks of their employment [51]. Not only can the experienced doctor serve as a role model for the novice but this shadowing offers room for gradual exposure to more responsible and complex tasks.

Apart from these two aspects of ED staffing, efforts can be made to make working in the ED less complex. For instance, decision support tools can be implemented [21]. Posters or pocketbooks with information on common problems (e.g., antibiotic therapy) and automated pop-ups in electronic medical records alerting the doctors of the possibility of sepsis are other examples. In addition, a lean organization of the ED with quiet working stations, linear processes, and good team communication reduces multitasking, improves efficiency, and is less complex [52].

Efforts can also be made to make the novice doctor a member of the ED team. The novice works in an unknown environment, with unfamiliar and probably more experienced colleagues. All team members should be encouraged to participate in teamwork programs and a culture should be created of teamwork and learning [38]. The involvement of nurses (and prehospital professionals, like paramedics) in this team-building process is effective [53] and, in our opinion, logical.

Lastly, part of the organizational solutions includes the design of a feedback and quality control loop. Much can be learned from knowing the course of the disease and patient diagnosis and outcome [20].

## Conclusions

Acknowledging that novice doctors are currently underprepared for working in the highly demanding environment of an ED is essential. Novice doctors are exposed to stress and are at risk of burnout and insufficient job satisfaction. The patient is at risk of adverse outcomes and the care system is at risk of inefficiency. Preparation and training and a sound organization of the ED that address the gaps in preparedness can improve acute care for all.
